# Diverging Paths to the Self: The Distinct Psychological Roles of Nostalgia and Declinism in Personal Growth

**DOI:** 10.3390/bs15101388

**Published:** 2025-10-14

**Authors:** Zhuo Feng, Tim Wildschut, Constantine Sedikides, Jianning Dang

**Affiliations:** 1 Faculty of Psychology, Beijing Normal University, Beijing 100875, China; fengzhuo@mail.bnu.edu.cn; 2 School of Psychology, University of Southampton, Southampton SO17 1BJ, UK; r.t.wildschut@soton.ac.uk (T.W.); c.sedikides@soton.ac.uk (C.S.)

**Keywords:** nostalgia, declinism, personal growth, psychological measurement, motivation

## Abstract

We investigated how nostalgia and declinism, two distinct forms of past-oriented reflection, differentially relate to personal growth. In preregistered cross-sectional Study 1 (*N* = 810, Chinese sample), we measured nostalgia using three instruments: the Southampton Nostalgia Scale (SNS), the Nostalgia Inventory (NI), and the Personal Inventory of Nostalgic Experiences (PINE). Although SNS- and NI-based nostalgia were positively associated with personal growth and uncorrelated with declinism, PINE-based nostalgia was positively linked to declinism and unrelated to growth. Canonical correlation analysis further indicated conceptual overlap between PINE items and declinism. In experimental Study 2 (*N* = 360, Chinese sample), we manipulated nostalgia and declinism with the Event Reflection Task to test their causal effects. Compared to a control condition, nostalgia increased personal growth, but declinism reduced it. Collectively, the findings highlight the importance of measurement in nostalgia research and underscore the psychological distinction between nostalgia and declinism. Accurately capturing the tone and function of nostalgic reflection is essential to understanding its influence on the self, motivation, and well-being.

## 1. Introduction

Humans are motivated to pursue self-improvement (Deci & Ryan, 1985; Maslow, 1943; Sedikides & Hepper, 2009). Personal growth, broadly conceptualized as the motivation to improve oneself across multiple life domains (Robitschek, 1998; Robitschek et al., 2012), is a cornerstone of psychological well-being. It is linked to outcomes ranging from higher self-esteem, self-efficacy, life satisfaction, and psychological well-being to lower stress, anxiety, psychological distress, and depression (de Freitas et al., 2016; Weigold et al., 2020). Furthermore, individuals who interpret their life transitions in terms of growth report more satisfying transitions and personality development in general (Bauer & McAdams, 2004). Lastly, personal growth is associated with reduced self-discrepancies (both actual-ideal self and ought-ideal self-discrepancies; Hardin et al., 2003) and stronger career exploration processes (Robitschek & Cook, 1999).

But what fuels this forward-oriented motivation? Turning to the past may seem a surprising answer. Research has identified the past-oriented emotion of nostalgia as a powerful psychological resource that boosts the approach motivation necessary for growth (Huang et al., 2016; Stephan et al., 2014). However, not all reflection on the past is functionally equivalent. Declinism, the belief that the past was superior to the present, also evokes a wistful longing for what has been lost but is rooted in a gloomy view of the current world. Although they may appear similar, these two constructs are often conflated in the psychological literature, potentially obscuring their distinct theoretical and empirical contributions. In this article, we seek to clarify this ambiguity by examining two key issues: (1) whether nostalgia and declinism are differentially associated with, or exert distinct effects on, personal growth; and (2) whether the relation between nostalgia and personal growth depends on the specific measure of nostalgia used. To address these issues, we conducted two complementary studies, a cross-sectional and an experimental one, designed to disentangle the unique psychological functions of nostalgia and declinism.

### 1.1. Nostalgia

Nostalgia is defined as “an affectionate feeling you have for the past, especially for a particularly happy time” (Collins English Dictionary, 2023). It involves the fond recollection of cherished memories (e.g., graduations, family vacations, wedding anniversaries, Thanksgiving dinners), often accompanied by a sense of loss due to the irreversible passage of time (Abeyta et al., 2015; Wildschut et al., 2006; Yin et al., 2025). As such, nostalgia is an ambivalent emotion, blending positive affect, such as contentment and joy, with a subtle undertone of sadness (Hepper et al., 2012, 2021; Sedikides & Wildschut, 2016a). Yet, empirical studies and lay conceptions alike depict nostalgia as predominantly positive and imbued with personal meaning (Hepper et al., 2014, 2024; Sedikides & Wildschut, 2018). Indeed, individuals high in trait nostalgia or those experiencing experimentally evoked nostalgia rate their past selves favorably (Batcho, 1995; Osborn et al., 2020; Sedikides & Wildschut, 2024).

Nostalgia is also an identity-based emotion. In particular, it consolidates the self-concept by fostering self-continuity, that is, a sense of connection among one’s past, present, and future selves (Hong et al., 2021, 2022; Sedikides et al., 2023). Furthermore, nostalgia carries motivational implications. Nostalgic memories are not merely retrospective; they serve prospective functions by energizing and directing behavior toward personally meaningful goals (Sedikides et al., 2018; van Dijke et al., 2019). Individuals often draw on nostalgic recollections to reaffirm their values, maintain a sense of purpose, and bolster goal commitment in the face of challenges (Sedikides & Wildschut, 2016b, in press). This emotional experience promotes a positive appraisal of one’s current life circumstances and enhances optimism regarding future prospects (Cheung et al., 2013, 2016; Sedikides & Wildschut, 2020).

### 1.2. Declinism

Declinism is defined as “a belief that everything is gradually becoming less, worse, or lower” and “liking for things or objects from days of yore” (Cambridge Dictionary; https://dictionary.cambridge.org/dictionary/english/declinist, accessed on 1 October 2025). Like nostalgia, declinism evokes a longing for the past and a desire to return to it. However, unlike nostalgia, which centers on meaningful aspects of one’s own past, declinism reflects a broader, often cultural or societal perspective that regards the past as superior to the present (Stern, 1992). Specifically, declinism entails the belief that, with the passing of time, core elements of society, such as living standards, morality, altruism, and overall quality of life, have progressively deteriorated (Holbrook, 1993; Mastroianni & Gilbert, 2023; Protzko & Schooler, 2019). It frames the current era as deficient when contrasted with an idealized past, celebrating historical triumphs while lamenting modern shortcomings (Holbrook & Schindler, 1994; Showalter, 1990). In this way, declinism extends beyond a mere appreciation of antiquity. It is characterized by a disaffection with the present and a pessimistic outlook on the future, rooted in what Whissen (1989, p. 73) termed an “incurable thirst for the sense of escape.” The phenomenon underscores a perceived barrenness in contemporary life and often manifests in cultural, political, or generational discourses that idealize the past while casting doubt on present and future trajectories (Inglehart, 2018; Sharot, 2011).

Although nostalgia is associated with positive personal and interpersonal outcomes (Hepper & Dennis, 2023; Layous & Kurtz, 2023; Sedikides & Wildschut, 2019), declinism is likely to be associated with negative outcomes. As a case in point, declinism has been linked to decreased faith in human nature (Abrams, 1988; Batcho, 2013), interpersonal trust (Putnam, 2000; Twenge et al., 2014), and importance of social relationships (Showalter, 1990; Twenge et al., 2014) as well as with increased pessimism (Herman, 2009; Milojević, 2021).

### 1.3. Divergent Relations of Nostalgia and Declinism with Personal Growth: A Confirmatory Approach

Consistent with its motivational character, personal growth is defined as the deliberate and sustained effort to cultivate one’s personality and actively shape the trajectory of one’s life to improve oneself (Brandtstädter et al., 1999; Lerner & Walls, 1999; Robitschek et al., 2012). This orientation, also referred to as “the potential to … seek out optimal challenges” (Baldwin & Landau, 2014, p. 163), reflects a proactive engagement with the self and a commitment to continuous self-improvement (Weigold et al., 2013; Weigold & Robitschek, 2011).

We are interested in the relation, correlational or causal, between nostalgia and personal growth. Individuals conceptualize nostalgia as an approach-oriented rather than an avoidance-oriented emotion (van Tilburg et al., 2018). Approach motivation is “the impulse to go forward” (Harmon-Jones et al., 2013, p. 291) or “the energization of behavior by, or the direction of behavior toward, positive stimuli (objects, events, possibilities)” (Elliot, 2006, p. 111). It can be initiated by both trait (Gray & McNaughton, 2000) and state (Panksepp, 1998) processes. At the trait level, nostalgia is positively associated with approach motivation (Stephan et al., 2014, Studies 1–2), with the latter construct being operationalized in terms of the Behavioral Activation System scale (Carver & White, 1994), and, in particular, the Fun Seeking (e.g., “I will often do things for no other reason than that they might be fun”) and Drive (e.g., “I go out of my way to get things I want”) subscales. At the state level, experimentally induced nostalgia strengthens approach motivation (i.e., fun seeking and drive), measured with the state version of the Behavioral Activation System scale (Huang et al., 2016, Study 4; Stephan et al., 2014, Study 3).

Given that striving for personal growth constitutes a form of approach motivation, we contend that personal growth is positively linked with and galvanized by nostalgia. In supporting this view, the construct of nostalgia encompasses prototypical features indicative of growth. For example, laypersons spontaneously employ growth-related language, such as references to change and the future, when describing nostalgic experiences (Hepper et al., 2012, Studies 1–2). Also, indirect evidence supports this proposition. As Baldwin and Landau (2014) illustrated, nostalgia increases curiosity and exploratory tendencies, constructs closely linked to personal growth, when assessed with the Curiosity and Exploration Inventory (e.g., “I am the kind of person who embraces unfamiliar people, events, and places”; “I view challenging situations as an opportunity to grow and learn”; Kashdan et al., 2009) and the Exploration Inventory (e.g., “I would like to spend a semester studying abroad”; “I would like to try bungee jumping, skydiving, or other adventurous activities”; Green & Campbell, 2000). Based on the above literature review, we hypothesize that nostalgia is associated with, or galvanizes, personal growth (Hypothesis 1).

We were further interested in the relation, correlational or causal, between declinism and personal growth. As previously noted, declinism is linked to several personal and interpersonal costs, including diminished faith in humanity, reduced interpersonal trust, devaluation of social relationships, and a generally bleak worldview (Abrams, 1988; Milojević, 2021; Putnam, 2000; Twenge et al., 2014). It is plausible that the pervasive pessimism inherent in declinist beliefs extends to self-related outcomes, such as personal growth. Accordingly, we hypothesize that declinism is negatively associated with, or undermines, personal growth (Hypothesis 2).

### 1.4. Differentiating Among Nostalgia Measures: An Exploratory Approach

We hypothesized so far that nostalgia would be positively, whereas declinism would be negatively associated with personal growth. These relations, though, might be contingent on the scale used to measure nostalgia. We proceeded to explore this possibility.

The three most widely used scales to assess nostalgia (Wildschut et al., 2023) are the Southampton Nostalgia Scale (SNS; Sedikides et al., 2015; Wildschut & Sedikides, 2022b), the Nostalgia Inventory (NI; Batcho, 1995), and the Personal Inventory of Nostalgic Experiences (PINE; Newman et al., 2020). The SNS consists of seven items. Four items assess proclivity (e.g., “How prone are you to feeling nostalgic?”; 1 = *not at all*, 7 = *very much*) or frequency (e.g., “How often do you experience nostalgia?”; 1 = *very rarely*, 7 = *very frequently*) of nostalgic engagement, and three items assess the personal relevance of nostalgia (e.g., “How valuable is nostalgia for you?”; 1 = *not at all*, 7 = *very much*). The NI assesses the extent of nostalgizing for 18 persons, situations, or events from one’s youth (e.g., “my childhood toys,” “vacations I went on,” “my family house”; 1 = *not at all*, 7 = *very much*). Lastly, the PINE comprises four items: “How nostalgic do you feel?”, “To what extent do you feel sentimental for the past?”, “How much do you feel a wistful affection for the past?”, and “To what extent do you feel a longing to return to a former time in your life?” (1 = *not at all*, 7 = *very much*).

Each of these instruments operationalizes nostalgia in subtly different ways, potentially yielding divergent associations with psychological constructs such as personal growth. In particular, the SNS captures individual differences in nostalgia proneness or value assigned to the nostalgic experience, the NI emphasizes nostalgia for aspects of one’s youthful past, and (as the acronym indicates) the PINE focuses on the intensity of nostalgic reverie and longing for a return to one’s past. On the face of it, it appears that the SNS and NI are closer to the definition of nostalgia as “an affectionate feeling you have for the past, especially for a particularly happy time” (Collins English Dictionary, 2023), whereas the PINE, especially the last two items, is closer to the definition of declinism as “a belief that everything is gradually becoming less, worse, or lower” and “liking for things or objects from days of yore” (Cambridge Dictionary; https://dictionary.cambridge.org/dictionary/english/declinist, accessed on 1 October 2025). We tested the possibility in several ways in Study 1. Specifically, we examined whether (a) the PINE is more strongly correlated with declinism than the SNS and NI are; (b) the SNS and NI are positively linked to personal growth, whereas the PINE is unrelated to personal growth; and (c) these patterns are mostly due to the PINE’s last item (“To what extent do you feel a longing to return to a former time in your life?”) and its conceptual overlap with declinism. We additionally explored whether these patterns are partially due to the PINE’s third item as well (“How much do you feel a wistful affection for the past?”).

### 1.5. Overview

We conducted two studies. In cross-sectional Study 1, we assessed nostalgia (with the SNS, NI, or PINE), declinism, and personal growth to examine the associations of nostalgia and declinism with personal growth and whether nostalgia measures matter. In experimental Study 2, we induced nostalgia and declinism (vs. control) to test their differing causal influence on personal growth. We hypothesized that, whereas nostalgia would be positively associated with and have a facilitating influence on personal growth (Hypothesis 1), declinism would be negatively associated with and have a hindering influence on personal growth (Hypothesis 2). For exploratory purposes, we examined whether the link between nostalgia and personal growth would be contingent on the scale used for measurement.

We pre-registered Study 1 (https://aspredicted.org/3hnr-9ytw.pdf, accessed on 1 October 2025). Data and analysis code are available at https://osf.io/wea5u. We provided the research protocol in Supplementary Materials.

## 2. Study 1

In preregistered Study 1, we examined the roles of nostalgia and declinism in predicting personal growth. We hypothesized that nostalgia—measured with three scales—would be positively associated with personal growth, whereas declinism would exhibit a negative association. Additionally, we explored whether the hypothesized positive association between nostalgia and personal growth would emerge consistently across the different measures of nostalgia.

### 2.1. Method

#### 2.1.1. Participants and Design

We used a three-condition, between-subjects design, with each condition corresponding to one of the three nostalgia scales. We chose this approach to prevent potential interference among the scales that could arise in a within-subjects design. For each condition, an *N* = 250 would be needed to achieve stable correlations (Schönbrodt & Perugini, 2013). As preregistered, we aimed to recruit 810 Chinese participants via the online platform Credamo, that is, 270 participants per condition. The platform automatically excluded those who failed one of two attention checks. The final sample, *N* = 810 (534 women, 276 men), ranged in age from 18 to 68 years (*M* = 30.74, *SD* = 8.61), with 270 participants in each condition (SNS: 66.3% women, NI: 67.8% women, PINE: 63.7% women).

#### 2.1.2. Procedure and Measures

All participants completed a declinism scale, one nostalgia scale (out of three), and a personal growth scale. Specifically, we randomly assigned them to one of three conditions: SNS, NI, or PINE. In each condition, participants also completed a declinism scale. We counterbalanced the order of a given nostalgia measure (SNS, NI, or PINE) and the declinism scale. Finally, all participants filled out a personal growth scale. We provide more information below.

**Nostalgia.** One third of participants completed the 7-item SNS (α = 0.92). Another third completed the NI (α = 0.79). The final third of participants completed the PINE (α = 0.85).

**Declinism.** We measured declinism with the 8-item Holbrook (1993) scale. Four items indicate a declining societal trend (e.g., “We are experiencing a decline in the quality of life,” “Things used to be better in the good old days”), and four items indicate an improving societal trend (e.g., “History involves a steady improvement in human welfare,” “Modern business constantly builds a better tomorrow”; 1 = *strongly disagree*, 7 = *strongly agree*). After reverse-scoring the improving trend items, we averaged all responses to create a declinism index (α = 0.79). Higher scores reflect greater declinism. All participants completed the declinism scale.

**Personal Growth.** We measured personal growth with the 16-item Personal Growth Initiative Scale–II (Robitschek et al., 2012; 1 = *strongly disagree*, 7 = *strongly agree*). It consists of four subscales: Intentional Behavior (e.g., “I know steps I can take to make intentional changes in myself”), Using Resources (e.g., “I ask for help when I try to change myself”), Planfulness (e.g., “I set realistic goals for what I want to change about myself”), and Readiness for Change (e.g., “I can tell when I am ready to make specific changes in myself”). We created an index by averaging responses to all items (α = 0.93), with higher scores reflecting more personal growth.

### 2.2. Results and Discussion

#### 2.2.1. Associations Among Variables

In Table 1, we present correlations among variables within each condition. In Table 2, we display comparisons of nostalgia’s correlations with declinism and personal growth between conditions. Analyses revealed a divergent pattern of associations between measures of nostalgia, personal growth, and declinism across the different nostalgia scales. For both the SNS and the NI, nostalgia was positively associated with personal growth and unrelated to declinism. In contrast, the PINE demonstrated no association with personal growth but was positively associated with declinism. Notably, across all conditions, declinism was negatively associated with personal growth.

So far, the PINE exhibited a distinct correlational profile relative to the SNS and NI, particularly in its simultaneous association with increased declinism and reduced personal growth. One potential explanation for this discrepancy is that the PINE conflates nostalgia with declinist sentiment. Given the between-subjects design, we were unable to examine associations between PINE items and items from the other nostalgia scales. Nevertheless, we conducted a canonical correlation analysis between the four items of the PINE and the eight items assessing declinism to evaluate their potential overlap.

The analysis revealed a significant first canonical pair with a strong correlation coefficient of *r* = 0.52 (Wilks’ λ = 0.686, *F* [32, 953.05] = 5.29, *p* < 0.001) between the PINE and declinism variates. Specifically, the PINE items “How much do you feel a wistful affection for the past?” and “To what extent do you feel a longing to return to a former time in your life?” emerged as the primary contributors to the PINE variate, exhibiting the strongest item loadings (|*r*s| = 0.94 and 0.83, respectively). Notably, these same items also demonstrated the highest correlations with the declinism variate (|*r*s| = 0.49 and 0.43, respectively), indicating a substantial degree of conceptual and empirical overlap. For detailed results, see Supplementary Materials, Table S1. These findings suggest that counterfactual pining for the past, especially when involving wistfulness (i.e., “sadly pensive especially about something yearned for”; Collins English Dictionary, 2023), confounds nostalgia and perceptions of societal decline.

#### 2.2.2. Distinct Effects of Nostalgia and Declinism on Personal Growth Across Conditions

To examine whether the relation between nostalgia and personal growth varies by the way nostalgia is operationalized, we conducted multi-group path analyses using three different nostalgia measures: the SNS, the NI, and the PINE. We included nostalgia and declinism as parallel and correlated predictors, with personal growth as the outcome variable. We conducted model comparisons to evaluate whether constraining the nostalgia–personal growth path across conditions resulted in significant losses in model fit. We report model fit statistics in Table 3.

**Figure 1:**
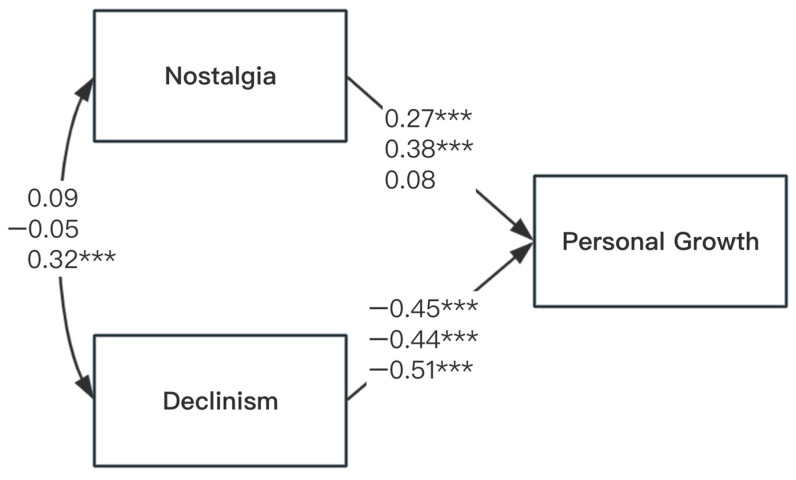
Differing Roles of Nostalgia and Declinism in Predicting Personal Growth in Study 1. *Note*. The three coefficients on each path represent path coefficients for the Southampton Nostalgia Scale (upper), Nostalgia Inventory (middle), and Personal Inventory of Nostalgic Experiences (lower) scale, respectively. Coefficients are all standardized. *** *p* < 0.001.

The reference model (M0), a fully saturated model in which all structural paths were freely estimated across the three measurement conditions (Figure 1), showed that nostalgia positively predicted personal growth in the SNS and NI conditions but had no predictive relation in the PINE condition. Consistently across all three conditions, declinism was negatively associated with personal growth.

In Model 1 (M1), we constrained the nostalgia–personal growth path to be equal across all three measurement conditions. This model fit the data significantly worse than M0 (Δχ^2^ = 34.80, Δdf = 2, *p* < 0.001), suggesting substantial variability in the association of nostalgia with personal growth depending on the nostalgia measure. In Model 2 (M2), we constrained the path to be equal across SNS and NI. M2 also fit significantly worse than M0 (Δχ^2^ = 22.23, Δdf = 1, *p* < 0.001), indicating that nostalgia had a stronger positive association with personal growth in the NI condition than in the SNS condition. In Model 3 (M3), we constrained the path across the SNS and PINE conditions, again resulting in poorer model fit (Δχ^2^ = 4.47, Δdf = 1, *p* = 0.034), suggesting that nostalgia was more strongly associated with personal growth in the SNS condition than in the PINE condition. Finally, Model 4 (M4), which constrained the path across the NI and PINE conditions, also demonstrated a significant loss of fit (Δχ^2^ = 34.80, Δdf = 1, *p* < 0.001), further supporting that the NI condition showed a more robust positive relation between nostalgia and personal growth than the PINE condition.

Collectively, these model comparisons indicate that the association between nostalgia and personal growth significantly differed across the three measurement conditions. The strongest positive association emerged in the NI condition, followed by a weaker positive association in the SNS condition, and no significant association in the PINE condition. These findings suggest that the operationalization of nostalgia meaningfully influences its association with growth-related outcomes.

### 2.3. Summary

In Study 1, we obtained empirical support for the distinct roles of nostalgia and declinism in predicting personal growth, underscoring the importance of treating these constructs as theoretically and empirically separable. Moreover, results revealed divergent profiles across nostalgia measures: nostalgia as assessed by the PINE was more closely aligned with declinist sentiment than nostalgia assessed via the SNS or NI. These differences in construct validity have important implications for how nostalgia is conceptualized and measured in psychological research.

## 3. Study 2

In Study 2, we built on the findings of Study 1 by experimentally testing the causal impact of nostalgia and declinism on personal growth. Specifically, we examined whether inducing nostalgia (vs. control) would lead to higher personal growth and whether inducing declinism (vs. control) would lead to lower personal growth.

### 3.1. Method

#### 3.1.1. Participants and Design

We employed a three-condition, between-subjects design (nostalgia, declinism, and control). Our focal interest was on the effects of nostalgia (vs. control) and declinism (vs. control) on personal growth. Based on the effect sizes from our Study 1 regression models (*R*^2^ = 0.088 for the nostalgia association and *R*^2^ = 0.185 for the declinism association), power analyses indicated that a sample size of *n* = 42 per condition and *n* = 19 per condition, respectively, would be required to achieve 80% power at an alpha level of 0.05. Given that this is the first experimental investigation on the topic, we opted to conservatively recruit, via Credamo, 360 Chinese participants (256 women, 104 men) ranging in age from 18 to 67 years (*M* = 30.88, *SD* = 8.11). We achieved a balanced design (i.e., *n* = 120 per condition). The platform excluded those who failed one of two attention checks. A sensitivity power analysis indicated that this sample size provided 80% statistical power to detect a main effect of condition with an effect size of η^2^ = 0.026. For comparison, in Study 1, the unique associations of nostalgia (assessed by three scales) and declinism with personal growth corresponded to partial *R*^2^ values ranging from 0.032 to 0.209.

#### 3.1.2. Procedure and Materials

**Nostalgia and Declinism Manipulation.** We manipulated nostalgia and declinism using a variant of the Event Reflection Task (Sedikides et al., 2015; Wildschut & Sedikides, 2025). Participants in the nostalgia condition recalled a nostalgia event in their lives, summarized its gist with four keywords, and then wrote a brief description of it. Participants in the declinism and control conditions followed the same protocol, reflecting instead on a declinist or an ordinary life event. Subsequently, all participants completed two 3-item manipulation checks, presented in counterbalanced order. The nostalgia check included the items “Right now, I am feeling quite nostalgic,” “Right now, I am having nostalgic thoughts,” and “I feel nostalgic at the moment” (α = 0.91). The declinism check included the items “Things used to be better in the good old days,” “Products are getting shoddier and shoddier,” and “I am experiencing a decline in the quality of life” (α = 0.75). We conducted an exploratory factor analysis of all six items with varimax rotation. We extracted two factors, with three nostalgia items loading on one factor (factor loadings > 0.881) and three declinism items loading on another factor (factor loadings > 0.683). The results of the exploratory factor analysis reinforce the conceptual distinctiveness of nostalgia and declinism.

**Personal Growth.** We assessed personal growth using five items that exhibited the highest loadings (all > 0.728) in an exploratory factor analysis of the Personal Growth Initiative Scale–II administered in Study 1. These items were as follows: “I set realistic goals for what I want to change about myself,” “I know how to make a realistic plan in order to change myself,” I know how to set realistic goals to make changes in myself,” “I know steps I can take to make intentional changes in myself,” and “I look for opportunities to grow as a person.” With this item selection, we aimed to reduce redundancy. We averaged responses to create a composite index of personal growth (α = 0.81).

### 3.2. Results

#### 3.2.1. Manipulation Check

To assess the effectiveness of our manipulations, we conducted one-way analyses of variance on the nostalgia and declinism manipulation check scores. For nostalgia, scores significantly differed across conditions, *F*(2, 357) = 43.65, *p* < 0.001, η^2^_p_ = 0.196, 90% CI [0.137, 0.254]. Participants in the nostalgia condition (*M* = 6.16, *SD* = 0.63) reported greater state nostalgia than those in the control (*M* = 4.80, *SD* = 1.50), *t*(357) = 9.23, *p* < 0.001, *d* = 1.19, 95% CI [0.94, 1.45] and declinism (*M* = 5.67, *SD* = 1.14), *t*(357) = 3.33, *p* < 0.001, *d* = 0.43, 95% CI [0.18, 0.68] conditions. Participants in the declinism condition also reported greater state nostalgia than those in the control condition, *t*(357) = 5.89, *p* < 0.001, *d* = 0.76, 95% CI [0.51, 1.02]. These results confirm the effectiveness of the nostalgia induction.

For declinism, scores also significantly differed across conditions, *F*(2, 357) = 36.63, *p* < 0.001, η^2^_p_ = 0.170, 90% CI [0.113, 0.227]. Participants in the declinism condition (*M* = 4.85, *SD* = 1.13) endorsed higher state declinism than those in the control (*M* = 3.56, *SD* = 1.27), *t*(357) = 8.49, *p* < 0.001, *d* = 1.10, 95% CI [0.84, 1.35] and nostalgia (*M* = 4.35, *SD* = 1.12), *t*(357) = 3.28, *p* = 0.001, *d* = 0.42, 95% CI [0.17, 0.68] conditions. Additionally, participants in the nostalgia condition reported higher state declinism than those in the control condition, *t*(357) = 5.24, *p* < 0.001, *d* = 0.68, 95% CI [0.42, 0.93]. These results support the effectiveness of the declinism induction.

#### 3.2.2. Effects of Nostalgia and Declinism on Personal Growth

**Figure 2:**
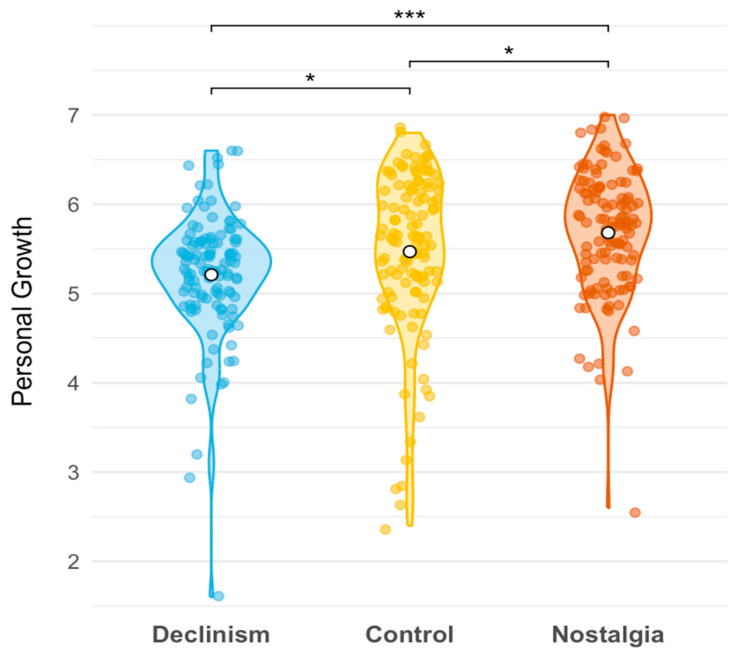
Personal Growth Across Conditions in Study 2. *Note*. Dots depict jittered individual data points. * *p* < 0.05. *** *p* < 0.001.

As shown in Figure 2, personal growth scores varied significantly across conditions, *F*(2, 357) = 11.22, *p* < 0.001, η^2^_p_ = 0.059, 90% CI [0.023, 0.100]. Participants in the nostalgia condition (*M* = 5.68, *SD* = 0.69) reported more personal growth than those in the control (*M* = 5.47, *SD* = 0.92), *t*(357) = 2.13, *p* = 0.034, *d* = 0.27, 95% CI [0.02, 0.53] and declinism (*M* = 5.21, *SD* = 0.69), *t*(357) = 4.73, *p* < 0.001, *d* = 0.61, 95% CI [0.36, 0.86] conditions. Furthermore, participants in the control condition reported more personal growth than those in the declinism condition, *t*(357) = 2.59, *p* = 0.010, *d* = 0.34, 95% CI [0.08, 0.59].

As noted above, participants in the nostalgia condition reported higher state declinism than those in the control condition. Thus, one possibility is that the observed increase in personal growth in the nostalgia (vs. control) condition was attributable to heightened state declinism. However, the negative correlation between state declinism and personal growth after controlling for state nostalgia (*r*_partial_ = −0.18, *p* < 0.001) rules this out. Similarly, as previously noted, participants in the declinism condition reported greater state nostalgia than those in the control condition. This raised the possibility that the observed decrease in personal growth in the declinism (vs. control) condition was due to increased state nostalgia. However, the positive correlation between state nostalgia and personal growth after controlling for state declinism (*r*_partial_ = 0.16, *p* = 0.002) rules this out.

## 4. General Discussion

We examined the distinct roles of nostalgia and declinism in shaping personal growth, a key psychological construct reflecting self-directed development and goal pursuit. Across two complementary studies, one correlational and one experimental, we tested the hypotheses that nostalgia is positively associated with, or promotes, personal growth, whereas declinism is negatively associated with, or reduces, it. Furthermore, we investigated whether the strength and direction of the nostalgia–growth association depend on how nostalgia is measured.

### 4.1. Implications

Our work has several implications. Theoretically, we addressed the distinction between nostalgia and declinism by demonstrating their opposing roles in predicting personal growth. Past research has examined the effects of these two past-oriented emotions independently (Holbrook & Schindler, 1994; Leunissen et al., 2021), often overlooking their conceptual and functional overlap. We addressed this knowledge gap by directly comparing nostalgia and declinism in both correlational and experimental contexts. Their divergent associations with personal growth, a construct often framed in terms of approach motivation and proactive self-regulation (Baldwin & Landau, 2014; Robitschek et al., 2012; Weigold et al., 2013), underscore their distinct functions: Although both are past-oriented, nostalgia invigorates a forward-looking and self-expansive orientation, whereas declinism anchors individuals to a bleak narrative of irreversible loss and deterioration (Dang et al., 2024, 2025; Sedikides & Wildschut, 2023; Wildschut & Sedikides, 2022a).

Methodologically, a critical contribution of our work lies in highlighting the importance of measurement specificity in nostalgia research. Although researchers have used a variety of scales to assess nostalgia (Wildschut & Sedikides, 2022b; Wildschut et al., 2023), they have frequently operated under the assumption that these instruments capture a common underlying construct. A limitation of this approach is the lack of systematic investigation into whether these measures conflate nostalgia with related, yet functionally distinct, constructs such as declinism. Seeking to clarify this issue, we undertook a comparative analysis of the three most widely used nostalgia scales. The Study 1 findings indicated that the three scales are not equivalent. The SNS and NI, both of which emphasize fondness for personally meaningful past events, were positively associated with personal growth and unrelated to declinism. In contrast, the PINE exhibited no association with personal growth and a significant positive association with declinism. Canonical correlation analysis revealed that two items on the PINE (those assessing “wistful affection for the past” and “longing to return to the past”) were primarily responsible for confounding nostalgic reverie with societal pessimism. These findings raise questions about the construct validity of the PINE while illustrating how lack of measurement precision can produce contradictory results. Our results thus encourage a reevaluation of nostalgia measurement, emphasizing the need to distinguish between self-relevant, meaning-infused recollections and more diffuse, culturally tinged longings for an idealized past.

Practically, these findings have relevance for interventions aimed at promoting psychological resilience and well-being. Therapeutic or educational approaches that harness nostalgic reflection, especially when guided toward affirming values and long-term goals, may foster growth-related outcomes. Conversely, addressing declinist narratives, particularly in aging populations or politically disaffected groups, may mitigate the demotivating effects of future pessimism.

### 4.2. Limitations and Future Directions

Several limitations warrant consideration and may direct future research. First, the implications of distinguishing between nostalgia and declinism for other psychological constructs remain unclear. For example, nostalgia marketing is widespread (Chen et al., 2014), and organizations that employ nostalgic cues (e.g., décor, themes) should carefully consider this distinction. Given their opposing effects on personal growth, nostalgia and declinism may also exert contrasting influences on consumer behaviors such as purchase intentions. Future research should therefore examine their impact on other outcomes, including consumer decisions and prosociality.

Second, the present article did not fully explore the underlying mechanisms of the nostalgia/declinism−growth link. Follow-up research should explore the mechanisms through which nostalgia promotes personal growth, such as increases in meaning in life (Sedikides & Wildschut, 2018), authenticity (Kelley et al., 2022), future self-continuity (Hong et al., 2024), and positive affect, as well as the mechanisms through which declinism hampers growth, such as negative affect.

The final set of limitations pertains to our experimental design and sample composition. Although Study 2 employed experimental methodology, the induction procedures may not have fully captured the complexity of naturally occurring experiences of nostalgia and declinism. Moreover, the observed effects may vary across cultural contexts and by gender, given the disproportionate number of women in our samples. This imbalance reflects, in part, the demographics of the Credamo platform, where the gender ratio typically skews toward women (approximately 1:1.5 to 1:2, women to men), a pattern observed across most studies recruiting from this source. Future research should replicate these findings using alternative induction methods, culturally diverse samples, and more balanced gender distributions.

### 4.3. Concluding Remarks

Our work demonstrates that nostalgia and declinism, while both rooted in the past, have divergent implications for personal growth. Nostalgia, particularly when measured in a way that foregrounds meaningful and fond past experiences, serves as a motivational resource, energizing future-oriented behavior. Declinism, by contrast, is marked by disengagement and dormancy. Importantly, these outcomes hinge on how nostalgia is conceptualized and measured. As interest in nostalgia continues to grow, it is increasingly important to approach its measurement with precision and strong theoretical grounding.

## Figures and Tables

**Table 1:** Zero-Order Correlations Among Variables in Study 1.

Condition	Variable	*M* (*SD*)	2	3	4	5
SNS (*n* = 270)	1. Nostalgia	5.00 (1.16)	0.09	0.22 ***	0.03	−0.11
	2. Declinism	3.13 (0.82)		−0.43 ***	−0.09	−0.04
	3. Personal Growth	5.66 (0.67)			0.13 *	−0.09
	4. Age	31.09 (8.24)				−0.13 *
	5. Gender	−				
NI (*n* = 270)	1. Nostalgia	5.23 (0.61)	−0.05	0.40 ***	0.06	−0.03
	2. Declinism	3.23 (0.76)		−0.46 ***	−0.09	0.02
	3. Personal Growth	5.54 (0.78)			0.17 **	−0.06
	4. Age	30.21 (8.43)				0.05
	5. Gender	−				
PINE (*n* = 270)	1. Nostalgia	4.63 (1.21)	0.32 ***	−0.08	−0.04	−0.04
	2. Declinism	3.17 (0.81)		−0.49 ***	−0.11	0.15 *
	3. Personal Growth	5.56 (0.76)			0.12 *	−0.19 **
	4. Age	30.91 (9.14)				−0.14 *
	5. Gender	−				

*Note*. SNS = Southampton Nostalgia Scale; NI = Nostalgia Inventory; PINE = Personal Inventory of Nostalgic Experiences. Gender coding: 1 = man, 2 = woman. * *p* < 0.05. ** *p* < 0.01. *** *p* < 0.001.

**Table 2:** Association Comparisons of Different Measures of Nostalgia with Other Variables in Study 1.

Correlation of Nostalgia with	Condition			Difference		
	SNS	NI	PINE	|*Z*_SNS-PINE_|	|*Z*_NI-PINE_|	|*Z*_SNS-NI_|
Declinism	0.09	−0.05	0.32 ***	2.79 **	4.41 ***	1.62
Personal Growth	0.22 ***	0.40 ***	−0.08	3.51 ***	5.82 ***	2.31 *

*Note*. SNS = Southampton Nostalgia Scale; NI = Nostalgia Inventory; PINE = Personal Inventory of Nostalgic Experiences. * *p* < 0.05. ** *p* < 0.01. *** *p* < 0.001.

**Table 3:** Model Comparisons Between the Unconstrained Model and Constrained Models in Study 1.

Model No.	χ^2^ (*df*)	*p*	RMSEA	CFI	TLI	SRMR
M0	0 (0)	0	0	1	1	0
M1	34.80 (2)	<0.001	0.246	0.888	0.498	0.047
M2	22.23 (1)	<0.001	0.280	0.928	0.350	0.039
M3	4.47 (1)	0.034	0.113	0.988	0.894	0.018
M4	34.80 (1)	<0.001	0.354	0.885	−0.035	0.047

*Note*. M0: the saturated model. M1: the model constraining the path between nostalgia and personal growth to be equal across three conditions. M2: the model constraining the path to be equal in the Southampton Nostalgia Scale and Nostalgia Inventory conditions. M3: the model constraining the path to be equal in the Southampton Nostalgia Scale and Personal Inventory of Nostalgic Experiences conditions. M4: the model constraining the path to be equal in the Nostalgia Inventory and Personal Inventory of Nostalgic Experiences conditions.

## Data Availability

All data and analysis code are available via the Open Science Framework (OSF) at https://osf.io/wea5u.

## Supplementary Materials

The following supporting information can be downloaded at: https://www.mdpi.com/article/10.3390/bs15101388/s1. Table S1: Results of Canonical Correlation Analyses in Study 1.
