# Supplementary material for: Diverging Paths to the Self: The Distinct Psychological Roles of Nostalgia and Declinism in Personal Growth
Source: Behav Sci (Basel). 2025 Oct 14;15(10):1388. doi: 10.3390/bs15101388 (PMC12561087; doi:10.3390/bs15101388)
Supplement: Supplementary file 1 [file behavsci-15-01388-s001.zip › behavsci-3763940-supplementary.pdf]

# SUPPLEMENTAL MATERIALS

## Diverging Paths to the Self:

## The Distinct Psychological Roles of Nostalgia and Declinism in Personal Growth

**MEASURES AND STIMULUS MATERIALS** .......................................................................... 2

**SUPPLEMENTAL ANALYSES** ......................................................................................... 8

# MEASURES AND STIMULUS MATERIALS

## Study 1

### Southampton Nostalgia Scale (SNS)

-How valuable is nostalgia for you? (1 = not at all, 7 = very much)

-How important is it for you to bring to mind nostalgic experiences? (1 = not at all , 7 = very much)

-How significant is it for you to feel nostalgic? (1 = not at all, 7 = very much)

-How prone are you to feeling nostalgic? (1 = n at all, 7 = very much)

-How often do you experience nostalgia? (1 = very rarely, 7 = very frequently)

-Generally speaking, how often do you bring to mind nostalgic experiences? (1 = very rarely, 7 = very frequently)

-Specifically, how often do you bring to mind nostalgic experiences? (Please check one.)

_____ Once or twice a year

_____ Once every couple of months

_____ Once or twice a month

_____ Approximately twice a week

_____ Approximately once a week

_____ Three to four times a week

_____ At least once a day

### Batcho's Nostalgia Inventory (NI)

Please indicate how much you are nostalgic for each of the 18 persons, situations, or events below from when you were younger (1 = not nostalgic at all, 7 = very nostalgic). The best answer is what you feel is true in general.

-my family

-vacations I went on

-places

-music

-someone I loved

-my friends

-things I did

-my childhood toys

-the way people were

-feelings I had

-my school

-having someone to depend on

-not having to worry

-the way society was

-my pets

-not knowing sad or evil things

-TV shows, movies

-my family house

**Personal Inventory of Nostalgic Experiences (PINE) Scale**

-How nostalgic do you feel? (1 = not at all, 7 = very much)

-To what extent do you feel sentimental for the past? (1 = not at all, 7 = very much)

-How much do you feel a wistful affection for the past? (1 = not at all, 7 = very much)

-To what extent do you feel a longing to return to a former time in your life? (1 = not at all, 7 = very much)

**Holbrook (1993) Declinism Scale**

-Things used to be better in the good old days.

-They don't make them like they used to.

-Products are getting shoddier and shoddier.

-We are experiencing a decline in the quality of life.

-History involves a steady improvement in human welfare. [reverse-scored]

-Technological change will insure a brighter future. [reversed-scored]

-Steady growth in GNP has brought increased human happiness. [reversed-scored]

-Modern business constantly builds a better tomorrow. [reversed-scored]

**Personal Growth Initiative Scale–II** (PGIS-2; Robitschek et al., 2012) (1 = strongly disagree, 7 = strongly agree):

-I set realistic goals for what I want to change about myself.

-I can tell when I am ready to make specific changes in myself.

-I know how to make a realistic plan in order to change myself.

-I take every opportunity to grow as it comes up.

-When I try to change myself, I make a realistic plan for my personal growth.

-I ask for help when I try to change myself.

-I actively work to improve myself.

-I figure out what I need to change about myself.

-I am constantly trying to grow as a person.

-I know how to set realistic goals to make changes in myself.

-I know when I need to make a specific change in myself.

-I use resources when I try to grow.

-I know steps I can take to make intentional changes in myself.

-I actively seek out help when I try to change myself.

-I look for opportunities to grow as a person.

-I know when it's time to change specific things about myself.

## Study 2

### Nostalgia and Declinism Manipulation

*Nostalgia Condition*

A Nostalgic Event from Your Past

According to the Oxford Dictionary, ‘nostalgia’ is defined as a “a sentimental longing for one’s past” or as “feeling sentimental about a fond and valued memory from one’s personal past.”

Please think of a nostalgic event in your life. Specifically, try to think of a past event that makes you feel nostalgic. Bring this nostalgic experience to mind. Immerse yourself in the nostalgic experience. How does it make you feel?

Please write down four keywords relevant to this nostalgic event (i.e., words that describe the experience).

Keywords that describe my nostalgic experience:

________________________________________________________________________

________________________________________________________________________

Using the space provided below, for the next few minutes, we would like you to write about the nostalgic event. Immerse yourself into this nostalgic experience. Describe the experience and how it makes you feel.

We will time you while you do this, and tell you when you can stop imagining yourself experiencing the nostalgic event.

________________________________________________________________________

________________________________________________________________________

*Declinism Condition*

A Declinism Occasion from Your Past

According to the Oxford Dictionary, ‘declinism’ is defined as “the belief that a society or

institution is tending towards decline" or that "as time passes, living conditions, morality, altruism, quality of life, the youth, or society in general are deteriorating."

Please think of a declinism experience in your life. Specifically, try to think of a past occasion in which you experienced a sense of decline. Bring this declinism experience to mind. Immerse yourself in the declinism experience. How does it make you feel?

Please write down four keywords relevant to this declinism experience (i.e., words that describe the experience).

Keywords that describe my declinism experience:

____________________________________________________________

____________________________________________________________

Using the space provided below, for the next few minutes, we would like you to write about the declinism experience. Immerse yourself into this declinism experience. Describe the experience and how it makes you feel.

We will time you while you do this, and tell you when you can stop imagining yourself experiencing the declinism occasion.

____________________________________________________________

____________________________________________________________

*Control Condition*

An Ordinary Event from Your Past

This is a study on autobiographical memory—that is, on your memory about your past.

Please think of an ordinary event in your life. Specifically, try to think of a past event that is ordinary. Bring to mind an objective record of this event and think it through as though you were a scientist or historian recording factual details (e.g., who did what, in what order). Immerse yourself in the ordinary experience. How does it make you feel?

Please write down four keywords relevant to ordinary autobiographical event (i.e., words that describe the experience).

Keywords that describe my ordinary experience:

___

___

Using the space provided below, for the next few minutes, we would like you to write about this ordinary event. Write a purely objective, factual, and detailed account (e.g., like a scientist or historian would do). Describe the experience and how it makes you feel.

We will time you while you do this, and tell you when you can stop imagining yourself experiencing the ordinary event.

___

___

How are you feeling right now?

-Right now, I am feeling quite nostalgic. (nostalgia manipulation check)

-Right now, I am having nostalgic feelings. (nostalgia manipulation check)

-I feel nostalgic at the moment. (nostalgia manipulation check)

-Things used to be better in the good old days. (declinism manipulation check)

-Products are getting shoddier and shoddier. (declinism manipulation check)

-I am experiencing a decline in the quality of life. (declinism manipulation check)

**Personal Growth**

-I can tell when I am ready to make specific changes in myself.

-I know how to make a realistic plan in order to change myself.

-When I try to change myself, I make a realistic plan for my personal growth.

-I know how to set realistic goals to make changes in myself.

-I know when it's time to change specific things about myself.

## SUPPLEMENTAL ANALYSES

**Table S1**

*Results of Canonical Correlation Analyses in Study 1*

| Item | Weight on the first canonical variate | Structure correlation on representative canonical variate | Structure correlation on the other canonical variate |
|---|---|---|---|
| PINE 1 | 0.020 | -0.76 | -0.40 |
| PINE 2 | -0.159 | -0.69 | -0.36 |
| PINE 3 | -0.454 | **-0.94** | **-0.49** |
| PINE 4 | -0.191 | **-0.83** | **-0.43** |
| Declinism 1 | -0.203 | -0.56 | -0.29 |
| Declinism 2 | -0.601 | -0.89 | -0.47 |
| Declinism 3 | 0.006 | -0.37 | -0.19 |
| Declinism 4 | -0.013 | -0.39 | -0.20 |
| Declinism 5 | -0.028 | -0.15 | -0.08 |
| Declinism 6 | -0.082 | -0.30 | -0.16 |
| Declinism 7 | 0.295 | -0.11 | -0.06 |
| Declinism 8 | -0.312 | -0.42 | -0.22 |

*Note.* $N = 270$. The weight on the canonical variate represents the canonical coefficient, indicating the unique contribution of an original item to the canonical variate, while controlling for the other items in the same set. The structure correlation is the Pearson correlation ($r$) between an original item and a canonical variate.
